# Prevalence of antimicrobial resistance in fecal *Escherichia coli* and *Enterococcus* spp. isolates from beef cow-calf operations in northern California and associations with farm practices

**DOI:** 10.3389/fmicb.2023.1086203

**Published:** 2023-02-23

**Authors:** Celeste Morris, Devinda Wickramasingha, Essam M. Abdelfattah, Richard V. Pereira, Emmanuel Okello, Gabriele Maier

**Affiliations:** ^1^William R. Pritchard Veterinary Medical Teaching Hospital, School of Veterinary Medicine, University of California, Davis, Davis, CA, United States; ^2^Department of Population Health and Reproduction, School of Veterinary Medicine, University of California, Davis, Davis, CA, United States; ^3^Veterinary Medicine Teaching and Research Center, School of Veterinary Medicine, University of California, Davis, Davis, CA, United States; ^4^Department of Animal Hygiene, and Veterinary Management, Faculty of Veterinary Medicine, Benha University, Benha, Egypt

**Keywords:** antimicrobial resistance, beef cattle, *Escherichia coli*, *Enterococcus* spp., feces, ceftiofur, tetracycline, cow-calf operations

## Abstract

Antimicrobials are necessary for the treatment of bacterial infections in animals, but increased antimicrobial resistance (AMR) is becoming a concern for veterinarians and livestock producers. This cross-sectional study was conducted on cow-calf operations in northern California to assess prevalence of AMR in *Escherichia coli* and *Enterococcus* spp. shed in feces of beef cattle of different life stages, breeds, and past antimicrobial exposures and to evaluate if any significant factors could be identified that are associated with AMR status of the isolates. A total of 244 *E. coli* and 238 *Enterococcus* isolates were obtained from cow and calf fecal samples, tested for susceptibility to 19 antimicrobials, and classified as resistant or non-susceptible to the antimicrobials for which breakpoints were available. For *E. coli*, percent of resistant isolates by antimicrobial were as follows: ampicillin 100% (244/244), sulfadimethoxine 25.4% (62/244), trimethoprim-sulfamethoxazole 4.9% (12/244), and ceftiofur 0.4% (1/244) while percent of non-susceptible isolates by antimicrobial were: tetracycline 13.1% (32/244), and florfenicol 19.3% (47/244). For *Enterococcus* spp., percent of resistant isolates by antimicrobial were as follows: ampicillin 0.4% (1/238) while percent of non-susceptible isolates by antimicrobial were tetracycline 12.6% (30/238) and penicillin 1.7% (4/238). No animal level or farm level management practices, including antimicrobial exposures, were significantly associated with differences in isolate resistant or non-susceptible status for either *E. coli* or *Enterococcus* isolates. This is contrary to the suggestion that administration of antibiotics is solely responsible for development of AMR in exposed bacteria and demonstrates that there are other factors involved, either not captured in this study or not currently well understood. In addition, the overall use of antimicrobials in this cow-calf study was lower than other sectors of the livestock industry. Limited information is available on cow-calf AMR from fecal bacteria, and the results of this study serve as a reference for future studies to support a better understanding and estimation of drivers and trends for AMR in cow-calf operations.

## 1. Introduction

Antimicrobial therapy is critical for the treatment of bacterial infections in veterinary medicine; however, resistance to these treatments has been increasing for decades (Thanner et al., 2016) and, as a result, there are concerns about the efficacy of antimicrobials in food-producing animals. It has been well documented that use of antimicrobial drugs is associated with increases in antimicrobial resistant bacteria (Agga et al., 2016; Thanner et al., 2016), but increases in antimicrobial resistance genes (ARG) can also develop in the absence of antimicrobial drug use (Agga et al., 2016). There is consensus that applying pressure on a population of bacteria through antimicrobials will enrich ARGs within that population (Xiong et al., 2018); however, AMR cannot be attributed to exposure to antimicrobials alone (Berge et al., 2010).

Although *Enterococcus* spp. are part of the normal flora in the bovine gastrointestinal tract, there are reports of *Enterococcus* spp. as a causative agent of mastitis in cattle (Devriese et al., 1992; Bradley et al., 2007) and diarrhea in neonatal calves (Ok et al., 2009). *Enterococcus* has, however, been primarily used as a sentinel and potential source of AMR genes for other Gram-positive pathogens. *Enterococcus* spp. are known to be intrinsically resistant to many antibiotics and can develop and confer AMR status to other pathogens (Cameron and McAllister, 2016; Torres et al., 2018). Thus, *Enterococcus* spp. have the potential to cause disease and serve as sentinels for the status of AMR pathogens in an environment. *E. coli* is another component of the normal flora of the bovine enteric system with many strains that have varying pathogenicity. Various *E. coli* strains can cause mastitis or metritis in cows as well as neonatal diarrhea or septicemia in calves (Burvenich et al., 2003; Sheldon et al., 2010; Dubreuil et al., 2016). *E. coli* has also been used as an indicator of AMR prevalence in fecal bacteria and a potential source for transmission of ARGs to other Gram-negative organisms (Cameron and McAllister, 2016), as it acquires resistance easily and can inhabit many types of animals (Tadesse et al., 2012).

California is an important contributor to the U.S. beef industry, with approximately 590,000 beef cows and contributing $3.4 billion in cash receipts from total cattle and calf sales in 2015 (Saitone, 2020). In beef cow-calf operations in the U.S., calves are born and stay at the same location with their dam usually until 6–8 months of age, at which point they are weaned, removed from the dam, and are often placed in a group with animals of approximately the same age and/or size. At this stage of the beef production cycle, there are many sectors that can involve the movement and mixing of animals. Some calves may be moved directly from cow-calf operations to feedlots after weaning until they reach slaughter weight. Alternatively, if there is high forage availability, they may be moved temporarily to a stocker facility before ultimately finishing at a feedlot facility. The time spent in each sector, size of group, and management of the animals are highly variable and depend on many factors including geographic location, producer goals, and access to pasture and/or facilities. Adult cull cows from cow-calf operations often go to slaughter directly at the end of their productive life.

Rates for multidrug resistance (MDR) have previously been shown to be higher in California cattle from various types of beef production systems when compared to nearby states, Washington and Oregon (Berge et al., 2010). Additionally, many AMR studies thus far have focused on feedlot, stocker, and calf ranch operations, where there may be increased selection pressure on bacterial populations through antimicrobial therapy from treatment and/or prevention of disease that develops likely as a result of mixing of animals, transport, and stress (Cameron and McAllister, 2016; Noyes et al., 2016). A 2010 study found that the highest proportion of MDR *E. coli* isolates originated from calf ranches, followed by feedlots, while the least MDR was found in isolates from adult beef cows (Berge et al., 2010). Prophylactic or metaphylactic use of antibiotics and occurrence of disease requiring antibiotic treatment is less common in cow-calf operations than other operation types in the beef production chain (Noyes et al., 2016). There are far fewer studies investigating the levels of AMR that exist in cow-calf operations and not yet one that exclusively investigated the levels of AMR that exist on cow-calf operations in California. Nevertheless, characterization of AMR in cow-calf operations is essential for evaluating and understanding the contribution of the cow-calf sector to AMR in the beef industry, as well as whether specific management and antimicrobial use patterns may be associated with AMR during this production stage before the calves are moved to feedlots where higher selection pressures exist. Previous studies in cow-calf herds have indicated that management and operation-dependent factors can influence the presence of AMR in a group. Specifically, season of collection of samples for testing (spring calves show more AMR than fall calves) (Gow et al., 2008), age of animal (calves show more AMR than feeding cattle or adult cattle) (Yamamoto et al., 2013), and intensity of operation (more intensive operations have more AMR than less intensive operations) (Hille et al., 2017) have all been shown to be associated with increased AMR detection in beef operations.

As part of the ongoing effort on surveillance for AMR, the objective of this cross-sectional study conducted on cow-calf operations in northern California was to assess prevalence of AMR in *E. coli* and *Enterococcus* spp. in beef cattle of different life stages, pasture types, and antimicrobial drug exposure on a herd and individual level. The hypothesis was that the prevalence of AMR in fecal samples varies according to the age of the animal as well as the drugs commonly used in the treatment of sick animals on those operations. The results of this study may serve as reference for future studies on the prevalence of AMR genes in the population of cow-calf operations in California and lead to a better understanding of risk factors for shedding of fecal pathogens carrying ARGs. Further surveillance, risk assessment, and interpretation of these results will help to derive more informed decisions and directions for combatting AMR in the future.

## 2. Materials and methods

### 2.1. Farm selection

A convenience sample of beef cow-calf operations in northern California was enrolled in this study either through the network of University of California Cooperative Extension livestock advisors or as clients of the Veterinary Medical Teaching Hospital at the University of California, Davis. Enrollment criteria included farms with a geographic location in northern California and primary production sector as beef cow-calf. No restrictions were placed on the type of operation (organic, conventional, other), herd size, grazing practices, breed of beef cattle, or previous antimicrobial use. All experimental protocols regarding animal use were approved by the Institutional Animal Care and Use Committee (protocol #21174) at the University of California, Davis.

### 2.2. Animal selection

Fecal samples were collected between June 2019 and August 2020 from cows and/or calves on each farm as a convenience sample, by either the herd veterinarian, extension veterinarian, or cooperative extension livestock advisor. Fecal samples were collected from a combination of calves aged between 1 week to 1 year and adult cows aged between 2 and 10 years either from the rectum or from freshly voided manure (pasture samples) after the animal was observed to defecate. Number and life stage of animal samples was based on number of animals available for sampling and that could be observed defecating within an hour of observation at the time of farm visit with the goal of sampling 5 cows and 5 calves per farm. Individual animal identifier, age, life stage (calf or cow), and breed were recorded when available.

### 2.3. Fecal sample collection

Fecal samples were collected during a single visit to each farm. Individual disposable gloves were used for collection of each sample and samples were stored in individual 15 mL polypropylene sample tubes. Rectal samples were collected on 8 farms from the recto-anal junction with individual rectal palpation sleeves. Pasture samples were collected as fresh feces (approximately 30–50 g) *via* gloved hand from the top and center of a freshly voided fecal pat, where the individual animal was observed to defecate. Samples were transported on ice to the laboratory at the University of California, Davis where they were kept refrigerated at 4°C if culture could be performed within 48 h or stored at −80°C in tryptic soy broth with 25% glycerol.

### 2.4. Farm survey and data collection

At time of fecal sample collection, an in-person survey regarding management and production practices was conducted. Information was collected on herd size, breed(s), certification status (organic, natural, or any other specialty certified programs), whether any farm personnel were Beef Quality Assurance (BQA) certified (specialized cattle management and food safety certification program), type of pasture (irrigated vs. dryland), whether the farm incorporated feeding of byproducts, water trough cleaning practices, existence of a current veterinarian-client-patient relationship, and whether the farm had submitted samples in the previous 12 months to a diagnostic laboratory. The survey also included detailed questions regarding antibiotic practices on the farm, specifically use of antibiotics in feed, use of intramammary antibiotics, use of injectable antibiotics, practices related to injectable antibiotics including indication for treatment, method for determining treatment duration and dosage, information recorded regarding treatment, and specific antibiotic(s) used in each method listed above. Treatment with antimicrobials in the past 6 months before sample collection were recorded for all sampled animals based on ranch records, markings on treated animals, or rancher’s recollection of treatments.

### 2.5. Isolation of bacteria

Selective growth media, *E. coli* Chromoselect Agar B and Rapid Enterococci Chromoselect Agar, following manufacturer guidelines (MilliporeSigma, Merck KGaA, Darmstadt, Germany), were used for culture and isolation of the respective bacterial types as previously described (Abdelfattah et al., 2021). Briefly, each fecal sample was streaked on the respective selective media using sterile cotton tipped applicators (Puritan Medical Products Co LLC, Guilford, Maine, USA) and incubated at 44°C (*E. coli*) or 35°C (*Enterococcus* spp.) for 24 h. Both *E. coli* and *Enterococcus* colonies were identified by characteristic blue green colony types on the Chromoselect plates. Two discrete colonies of each bacterial type were selected and purified on 5% sheep blood agar plates (Biological Media Services, University of California, Davis). The pure colonies were stored in tryptic soy broth with 25% glycerol at −80°C until all farm sampling was complete.

### 2.6. Selecting isolates for antimicrobial susceptibility testing

**Figure 1:**
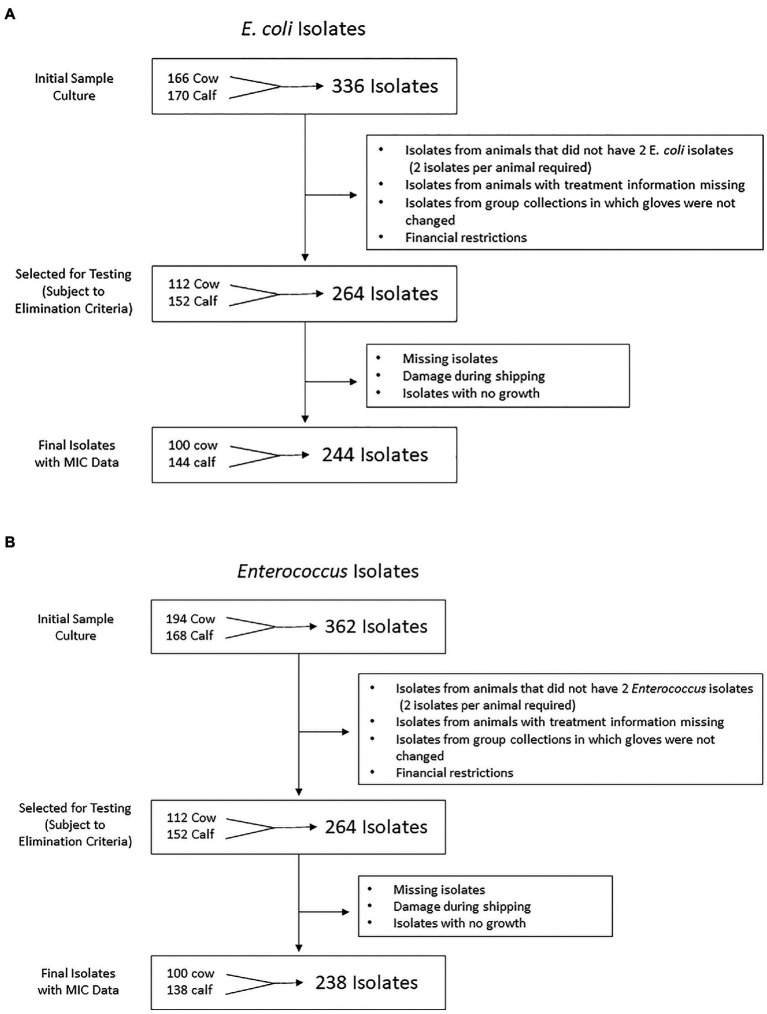
Flow chart representation of **(A)** *Escherichia coli* and **(B)** *Enterococcus* isolate numbers at each stage of the study from farm to antimicrobial susceptibility testing. 244 *E. coli* and 238 *Enterococcus* isolates were isolated from cow and calf fecal samples in a study on prevalence of antimicrobial resistance on cow-calf operations in northern California collected between July 2019 and August 2020.

After initial culture, a total of 698 bacterial isolates (362 *Enterococcus* spp., 336 *E. coli*) were stored. From these, 528 bacterial isolates were selected for antimicrobial susceptibility testing (264 *Enterococcus*, 264 *E. coli*). Exclusion criteria included (1) isolates from a farm where the rectal sleeve was accidentally not changed between samplings, (2) all fecal samples which did not yield at least 2 identifiable isolates for each bacterial type after two culture attempts, and (3) any samples with missing or unknown treatment information. Of the 528 selected, 482 (244 *E. coli* and 238 *Enterococcus* spp.) were selected for antimicrobial susceptibility testing. See Figures 1A,B for flow charts of the isolate selection process for *E. coli* and *Enterococcus* isolates, respectively.

### 2.7. Antimicrobial susceptibility testing

Antimicrobial susceptibility testing (AST) was conducted by the broth microdilution method using Sensititre™ system (Thermo Fisher Scientific Inc., Waltham, USA) against a panel of 19 antibiotics on a commercially available BOPO7F Vet Antimicrobial Susceptibility Testing Plate (Table 1). In the AST procedure, 1–5 colonies of *E. coli* or *Enterococcus* were resuspended in 5 mL of demineralized water and the cell suspension adjusted to optical density of 0.5 McFarland using a nephelometer. Next, 10 μl of *E. coli* or *Enterococcus* bacterial suspension was added to 11 mL Mueller-Hinton broth (Thermo Scientific, Remel Inc., KS, USA) and mixed by repeated inversion of the tube. Fifty microliters of inoculated Mueller-Hinton broth were dispensed into each well of the 96-well BOPO7F Vet plate using Sensititre™ Autoinoculator and the plates were incubated for 18–24 h at 35°C. The minimum inhibitory concentration (MIC) was read using Sensititre™ Vizion™ Digital MIC Viewing System (Thermo Fisher Scientific Inc., Waltham, USA). The MIC values (lowest concentration of antimicrobial drug that inhibited the growth of bacteria) was interpreted following the Clinical and Laboratory Standards Institute (CLSI) guidelines [Clinical and Laboratory Standards Institute (CLSI), 2020; Clinical Lab Standards Institute (CLSI), 2022]. Quality control steps included checking for bacterial growth and colony purity by plating 1 μL of the inoculated Mueller-Hinton broth on TSA with 5% sheep blood. Contaminated or no-growth inoculated samples were not read and repeated. In addition, quality control strains (*E. coli* ATCC 35218 and *E. coli* ATCC 25922 and *Enterococcus faecalis* ATCC 29212) were run weekly alongside the test samples.

**Table 1:** Antimicrobials included in the BOPO7F vet antimicrobial susceptibility testing plate, dilution ranges, and breakpoints used for evaluation of 244 *Escherichia coli* and 238 *Enterococcus* isolates (**μ**g/mL). The data was used to interpret the antimicrobial susceptibility test results and estimate the prevalence of antimicrobial resistant *E. coli* and *Enterococcus* in fecal samples from cow-calf operations in northern California collected between July 2019 and August 2022.

Antimicrobial class	Antimicrobial drugs	Dilution range	*E. coli* Breakpoints^a^			*Enterococcus* Breakpoints^a^		
			S	I	R	S	I	R
Penicillins	Ampicillin	0.25–16	≤0.03	0.06–0.12	≥0.25	≤8	.	≥16
Cephalosporins	Ceftiofur	0.25–8	≤2	4	≥8	.	.	.
Lincosamides	Clindamycin	0.25–16	.	.	.	.	.	.
Fluoroquinolones	Danofloxacin	0.12–1	.	.	.	.	.	.	Fluoroquinolones
	Enrofloxacin	0.12–2	.	.	.	.	.	.
Amphenicols	Florfenicol	0.25–8	≤4	8	≥16	.	.	.
Macrolides	Gamithromycin	1 to 8	.	.	.	.	.	.
Aminoglycosides	Gentamicin	1 to 16	.	.	.	.	.	.
Aminoglycosides	Neomycin	4 to 32	.	.	.	.	.	.
Penicillins	Penicillin	0.12–8	.	.	.	≤8	.	≥16
Aminocyclitols	Spectinomycin	8 to 64	.	.	.	.	.	.
Sulfonamides	Sulfadimethoxine	256^b^	≤256	-	≥512	.	.	.
Tetracyclines	Tetracycline	0.5–8	≤4	8	≥16	≤4	8	≥16
Pleuromutilins	Tiamulin	0.5–32	.	.	.	.	.	.
Macrolides	Tildipirosin	1–16	.	.	.	.	.	.
Macrolides	Tilmicosin	2–16	.	.	.	.	.	.
Folate pathway antagonist	Trimethoprim- sulfamethoxazole	2/38^b^	≤2/38	-	≥4/76	.	.	.
Macrolides	Tulathromycin	8–64	.	.	.	.	.	.
Macrolides	Tylosin	0.5–32	.	.	.	.	.	.

a Breakpoint abbreviations as follows: S, Susceptible; I, Intermediate, and R, Resistant. Adopted from VET01S (5th edition) [Clinical and Laboratory Standards Institute (CLSI), 2020] and M-100 (32nd edition) [Clinical Lab Standards Institute (CLSI), 2022] from Clinical and Laboratory Standards Institute.

b Only one dilution available for these antimicrobials.

### 2.8. Breakpoint determination

When available, antimicrobial susceptibility veterinary breakpoints from the Clinical Laboratory Standards Institute (CLSI) were used to interpret MIC results [Clinical and Laboratory Standards Institute (CLSI), 2020], while human CLSI breakpoints were used for bacterial-drug combinations without veterinary breakpoints [Clinical and Laboratory Standards Institute (CLSI), 2020; Clinical Lab Standards Institute (CLSI), 2022]. All breakpoints used in this study were for the bacterium indicated. For antimicrobials in which the BOPO7F Vet AST Plate dilutions included the established breakpoint, “resistant” status was assigned if the isolate grew in or beyond the breakpoint dilution (ampicillin, ceftiofur, sulfadimethoxine, and trimethoprim/sulfamethoxazole for *E. coli* and ampicillin for *Enterococcus*). For antimicrobials in which the testing plate included only dilutions below the established breakpoint, “non-susceptible” status was assigned and included isolates in the intermediate range according to CLSI guidelines or isolates that grew in the highest dilution available. Resistance or non-susceptible status was only assigned to antimicrobials for which breakpoints were available and for which *in-vivo* activity and antimicrobial spectrum were applicable. For antimicrobials that were assigned non-susceptible status (florfenicol and tetracycline for *E. coli* and penicillin and tetracycline for *Enterococcus*), it was not possible to establish resistance because the drug dilutions did not reach the threshold breakpoint; hence growth or no growth at or beyond the breakpoint could not be established. Antimicrobial breakpoints used and dilution ranges for the BOPO7F Vet AST Plate can be found in Table 1.

### 2.9. Statistical analysis

Data from the ranch survey, individual animal data, and AST results were entered into a spreadsheet (Microsoft Excel, version 16.43, Redmond, WA) and combined using a relational database (Microsoft Access, Version 2010, Redmond, WA). Descriptive statistics for ranch demographics and prevalence of resistance or non-susceptibility for antimicrobials with existing breakpoints were prepared. Univariable generalized linear mixed models with a logit link were prepared for the outcome of resistance or non-susceptibility status of isolates to each antimicrobial with available breakpoint data using the GLIMMIX procedure in SAS (SAS Version 7.15 HF7; SAS Institute, Cary, NC). A random effect was added to account for correlation between isolates from the same animal, since 2 isolates from each fecal sample were required for inclusion in the MIC analysis. A second random effect of farm with animal nested within farm was attempted but led to non-positive G matrices and not explored further. The independent variables were created from the questionnaire data on herd demographics, antimicrobial practices, treatment history, and management practices on the farm. Multivariable generalized linear mixed models were attempted by including all variables from the univariable analysis with *p* < 0.2.

A multiple factor analysis (MFA) was conducted for survey data and antimicrobial susceptibility testing results of the 244 *E. coli* isolates. MFA was conducted to reveal the most important variables that explain the variation in the data set (Pagès, 2002). The dataset consisted of 63 data variables which were organized into 6 groups based on relatedness as follows: (1) herd information: a group of 7 categorical variables specifying farm number, the location of farm, breed distribution, herd size, certification status (e.g., certified organic), type of pasture, and type of production; (2) sampled animals’ life stage and treatment history: a group of 7 categorical variables specifying sampled animal life stage, method of fecal sample collection (from rectum or pasture), date of fecal sample collection, whether animal was treated with antimicrobials, and antimicrobial used for treatment (tetracycline, tulathromycin, or florfenicol); (3) antimicrobial resistance group: a group of 8 variables describing AMR for *E. coli* (resistance to ceftiofur, florfenicol, sulfadimethoxine, and trimethoprim/sulfamethoxazole); AMR for *E. coli*; (4) farm antimicrobial use and disease treatment group: a group of 17 categorical variables describing the different injectable and intramammary antimicrobial drugs used in farms and type of treated diseases (respiratory, scours, foot rot, navel infections, wounds, metritis, and mastitis); (5) antimicrobial dosing and record keeping practices: a group of 12 variables describing methods used for determining treatment duration and dosage, and information recorded regarding antimicrobial treatment (e.g., information recorded after antimicrobial treatment such as date, dose, route, withdrawal, and/or product name); and (6) nutrition related factors: a group of 12 categorical variables specifying the provision of byproducts and mineral supplement to calves, and cows (e.g., does the farm feed mineral to preweaned or weaned calves or cows). The groups with loading weights of 0.5 or higher on the first two principal components were retained for interpretation (Hille et al., 2017). The percentage of variability contributed by each group of variables to the principal components and the correlation coefficients for the component variables within each group were estimated (Abdelfattah et al., 2019). Variables within each group with loading weights of ≥0.5 on the first two principal components were also retained for interpretation. The function MFA in FactoMiner package was used to perform the MFA on the dataset. The function *get_mfa_var(res.mfa)* was used to extract the results for the groups and variables. Hierarchical clustering was performed on the MFA principal coordinates using the principal component methods at the animal level (Husson et al., 2010). The identified clusters were described based on the variables that contributed the most to the data variability. Both MFA and hierarchical clustering were performed in R software using FactoMineR for the analysis and factoextra for data visualization (Lê et al., 2008). MFA analysis was not performed for *Enterococcus* data due to the limited number of resistant and non-susceptible isolates.

## 3. Results

### 3.1. Survey

A total of 18 cow-calf farms were surveyed and sampled during this study. General descriptive data including the major breed, herd size, pasture type, location, antimicrobial practices, and the number of injectable or oral antibiotics used on farm is shown in Table 2. Other management survey results of interest revealed that most farms (16/18, 89%) had at least one beef quality assurance certified employee, one farm (6%) fed byproducts, 7 farms (39%) had submitted samples to a diagnostic lab in the past year, and 17 (94%) had an established veterinarian-client-patient relationship.

**Table 2:** Descriptive survey data from all farms sampled in a study on prevalence of antimicrobial resistant *E. coli* and *Enterococcus* in fecal samples from cow-calf operations in northern California collected between July 2019 and August 2020.

	# Farms	Farm %
**Major breed (>60% of Herd)**
Angus^a^	10	55.56%
Crossbred^b^	7	38.89%
Other	1	5.56%
**Herd size**
<100	6	33.33%
100–249	5	27.78%
250–499	3	16.67%
>499	4	22.22%
**Pasture type**
>50% Dryland	10	55.56%
≥50% Irrigated Pasture	8	44.44%
**Production type**
Conventional	14	77.78%
Other^c^	4	22.22%
Location		
Coastal Range	5	27.78%
Central Valley	8	44.44%
North Central Valley	5	27.78%
**Antimicrobial practices**
Antimicrobials in Feed	0	0.00%
Intramammary Antimicrobials	1	5.56%
**Number of injectable or oral antimicrobials used**
0	2	11.11%
1 to 2	7	38.89%
3+	9	50.00%

a Can indicate either Red or Black Angus.

b Cross = Includes Angus cross, Hereford cross, Charolais cross.

c Includes Organic, Natural, No Hormone Treated Certified, and/or Age and Source Verified.

Oxytetracycline was the most common antimicrobial used on farm (used by 14 of 18 farms, or 78%), followed by tulathromycin (10/18, 56%), florfenicol (9/18, 50%), sulfas including sulfadimethoxine and sulfamethoxazole (4/18, 22%), penicillin (3/18, 17%), enrofloxacin (2/18, 11%), and ceftiofur (1/18, 6%). No farm reported using danofloxacin or ampicillin. Regarding the types of diseases that had been treated with antimicrobials in the past 12 months in any cattle on the farm, 13 farms (72%) reported treating infectious bovine keratoconjunctivitis (pinkeye), 13 (72%) reported treating bovine respiratory disease, 10 (56%) reported treating foot rot, 7 (39%) reported treating scours, 6 (33%) reported treating wounds, 5 (28%) reported treating navel infections, 3 (17%) reported treating metritis, and 2 (11%) reported treating mastitis. There were 2 farms (11%) that reported no antimicrobial use because no disease identified as needing treatment was observed during the past year. Only one farm (6%) had routine prophylactic use of antibiotics where all calves received an injection of oxytetracycline between 1 week and 1 month of age, and all farms that used antimicrobials recorded at least one form of information after antimicrobials were administered such as date, dose, route, withdrawal, and/or product name.

### 3.2. Isolate growth

In total, fecal samples were collected from 187 animals (104 cows, 83 calves) and plated for growth and recovery of *E. coli* and *Enterococcus* isolates. A total of 244 *E. coli* isolates and 238 *Enterococcus* isolates were recovered and tested for antimicrobial susceptibility using broth microdilution method. Of the 104 cow samples plated, 50 samples grew at least 2 isolates of *E. coli* and 50 samples grew at least 2 isolates of *Enterococcus*. Of the 83 calf samples plated, 72 samples grew at least 2 isolates of *E. coli* and 69 samples grew at least 2 isolates of *Enterococcus*. Details regarding the number of samples and resulting isolates can be found in Figure 1.

### 3.3. *Escherichia coli* antimicrobial susceptibility testing

The distribution of isolates within various drug dilutions tested for each antimicrobial can be found in Table 3. Resistance or non-susceptible data is only shown for those antimicrobials for which established breakpoints by CLSI were available, including ampicillin, ceftiofur, florfenicol, sulfadimethoxine, tetracycline, and trimethoprim-sulfamethoxazole (Table 1). Among the 244 *E. coli* isolates, 88/244 (36.07%) were resistant or non-susceptible to at least one antimicrobial excluding ampicillin, to which all isolates were resistant. Similarly, a large proportion of isolates showed antimicrobial resistance or non-susceptibility to sulfadimethoxine followed by trimethoprim-sulfamethoxazole, while the lowest proportion of isolates showed antimicrobial resistance to ceftiofur. More isolates were classified as non-susceptible to tetracycline than florfenicol. Neither univariable nor multivariable generalized linear mixed models revealed any statistically significant associations between any of the risk factors considered, including record of antimicrobial therapy with the same antimicrobial in the past 6 months, and resistance or non-susceptible isolate status. Although none of the farm-specific variables captured in this study were significantly associated with differences in resistance or non-susceptibility, there were numerical differences between farms in terms of their antimicrobial resistance profile for *E. coli* isolates. Specifically, the highest percentage of resistant or non-susceptible isolates for florfenicol (57%), tetracycline (57%), and trimethoprim-sulfamethoxazole (64%) at the farm level was found on Farm 6, which contributed 14 isolates. Interestingly, Farm 6 did not report the use of any antimicrobials on farm.

**Table 3:** Distribution of 244 *E. coli* isolates inhibited by various concentrations of select antimicrobials. Antimicrobial susceptibility was conducted on *E. coli* isolated from fecal samples during a cross-sectional study on prevalence of antimicrobial resistance to *E. coli* and *Enterococcus* from cow-calf operations in northern California between July 2019 and August 2020.

Antimicrobial drugs	% Resistant (Red) or non-susceptible (Blue)	Number of isolates within each MIC^a^ (μg/mL)										
		0.12	0.25	0.5	1	2	4	8	16	32	64	GAD**
Ampicillin*	100%		0	0	3	105	125	5	0			5
Ceftiofur*	0.41%		129	110	2	0	0	0				1
Clindamycin	***		0	0	0	0	0	0	0			244
Danofloxacin	***	241	2	0	0							1
Enrofloxacin	***	243	1	0	0	0						0
Florfenicol	19.26%		0	0	0	40	157	27				20
Gamithromycin	***				1	9	64	158				12
Gentamicin	***				244	0	0	0	0			0
Neomycin*	***						241	0	0	0		1
Penicillin	***	0	0	0	0	0	0	4				240
Spectinomycin*	***							23	207	13	0	0
Sulfadimethoxine****	25.41%											62
Tetracycline	13.11%			3	107	101	1	5				27
Tiamulin	***			0	0	0	0	0	9	28		207
Tildipirosin	***				0	14	171	59	0			0
Tilmicosin	***					0	1	0	2			241
Trimethoprim- sulfamethoxazole****	4.92%											12
Tulathromycin	***							158	84	1	0	1
Tylosin	***			0	0	0	0	0	0	0		244

a Minimum Inhibitory Concentration.

*Rows do not add up to 244 due to missing data points from plate reader errors.

**Number of isolates that grew in all available dilutions (GAD) of each antimicrobial drug.

***Clinical and Laboratory Standards Institute breakpoints not available for this antimicrobial.

****Antimicrobials for which only one dilution was available for testing.

| (vertical bar) Threshold for breakpoints (any isolates with MICs higher than the vertical bar are considered resistant).

Red highlighted areas: number or percent of isolates classified as resistant to each antimicrobial drug.

Blue highlighted areas: number or percent of isolates classified as non-susceptible to each antimicrobial drug.

Grey areas: specific MIC was not included in the dilution range for the listed antimicrobial drug.

### 3.4. *Enterococcus* antimicrobial susceptibility testing

The distribution of isolates within MICs tested for each antimicrobial can be found in Table 4. Resistance or non-susceptible data is only shown for those antimicrobials for which established CLSI breakpoints were available, including ampicillin, penicillin, and tetracycline (Table 1). Only a small proportion of the total 238 *Enterococcus* isolates, 35/238 (14.7%) were resistant or non-susceptible to at least one antimicrobial. Amongst all isolates tested, antimicrobial non-susceptibility was highest to tetracycline, followed by non-susceptibility to penicillin, and lowest resistance to ampicillin. Similar to the statistical models for the *E. coli* isolates, no significant associations between any of the risk factors and AMR status for *Enterococcus* isolates was found.

**Table 4:** Distribution of 238 *Enterococcus* isolates inhibited by various concentrations of select antimicrobials. Antimicrobial susceptibility was conducted on *Enterococcus* isolated from fecal samples during a cross-sectional study on prevalence of antimicrobial resistance to *E. coli* and *Enterococcus* rom cow-calf operations in northern California between July 2019 and August 2020.

Antimicrobial drugs	% Resistant (Red) or Non-Susceptible (Blue)	Number of isolates within each MIC^a^ (ug/mL)										
		0.12	0.25	0.5	1	2	4	8	16	32	64	GAD**
Ampicillin	0.42%		129	73	32	0	2	1	0			1
Ceftiofur	***		126	4	4	1	8	23				72
Clindamycin	***		152	5	5	4	15	34	19			4
Danofloxacin	***	2	2	17	67							150
Enrofloxacin	***	3	4	28	100	96						7
Florfenicol	***		0	36	76	58	64	4				0
Gamithromycin	***				177	13	18	16				14
Gentamicin	***				14	60	84	59	21			0
Neomycin	***						52	88	57	36		5
Penicillin*	1.68%	125	3	36	33	23	13	0				4
Spectinomycin*	***							1	19	45	171	0
Sulfadimethoxine****	***											233
Tetracycline	12.61%			161	43	4	0	1				29
Tiamulin	***			103	31	9	5	11	3	0		76
Tildipirosin	***				124	5	1	9	33			66
Tilmicosin	***					128	1	13	54			42
Trimethoprim- sulfamethoxazole****	***											5
Tulathromycin	***							183	21	29	5	0
Tylosin	***			128	1	49	50	7	0	0		3

a Minimum inhibitory concentration.

*Rows do not add up to 238 due to missing data points from plate reader errors.

** Number of isolates that grew in all available dilutions (GAD) of each antimicrobial drug.

*** Clinical and Laboratory Standards Institute breakpoints not available for this antimicrobial.

****Antimicrobials for which only one dilution was available for testing.

| (vertical bar) Threshold for breakpoints (any isolates with MICs higher than the vertical bar are considered resistant).

Red highlighted areas: number or percent of isolates classified as resistant to each antimicrobial drug.

Blue highlighted areas: number or percent of isolates classified as non-susceptible to each antimicrobial drug.

Grey areas: specific MIC was not included in the dilution range for the listed antimicrobial drug.

### 3.5. Multiple factor analysis

**Figure 2:**
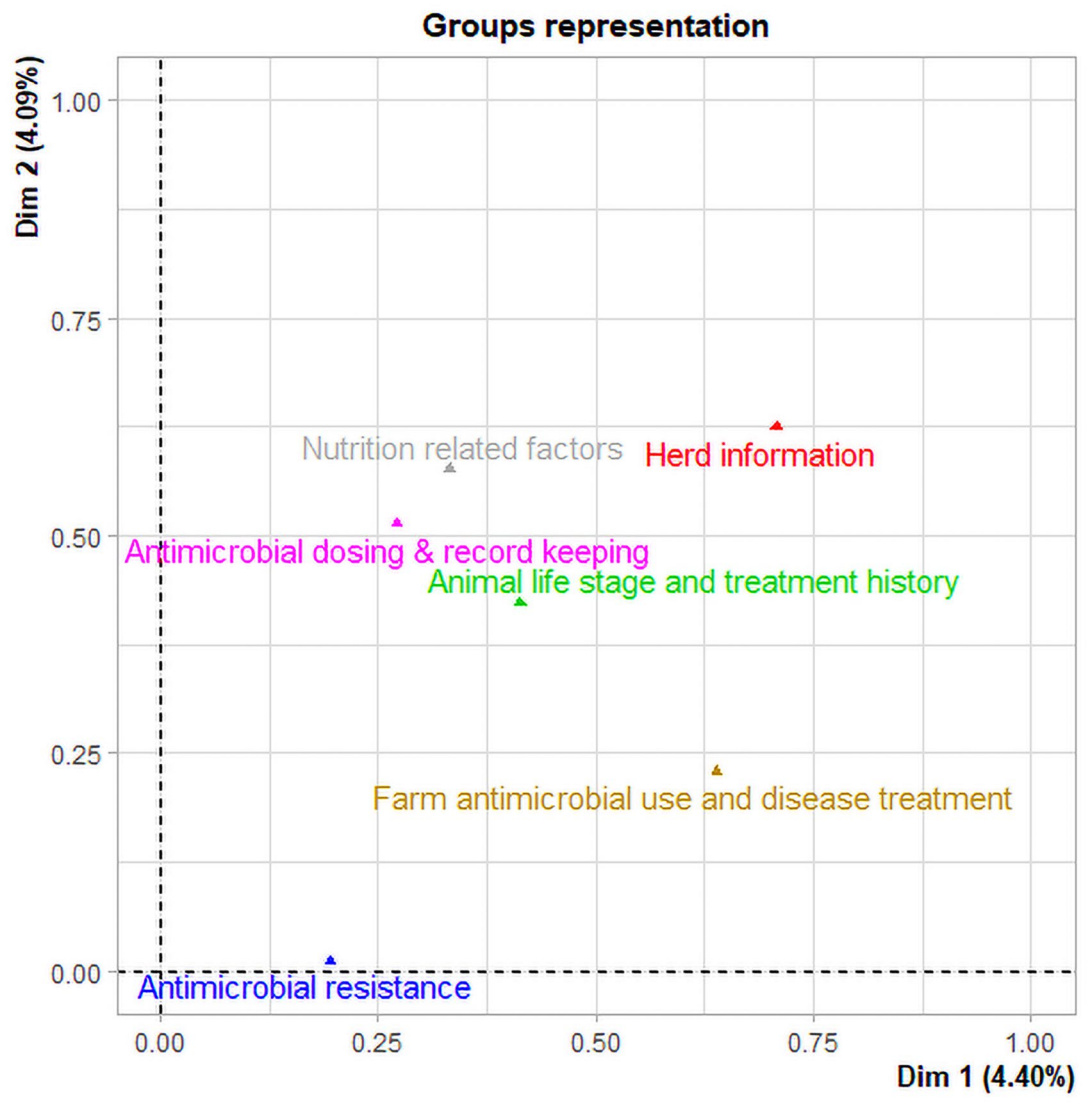
Multiple factor analysis of data collected from beef ranches in California in a cross-sectional study evaluating antimicrobial resistance and antimicrobial drug use in 18 cow-calf operations in northern California between July 2019 and August 2020. Two groups (Herd information and Farm antimicrobial use and disease treatment) have the highest coordinates indicating the largest contribution to the first dimension. In the second dimension, the three groups (Nutrition related factors, antimicrobial dosing and record keeping, and sampled Animal life stage and treatment history) have the highest coordinates indicating the largest contribution to the second dimension.

The first two principal component dimensions of the multiple factor analysis (MFA) explained approximately 8.5% of the variability in the data, i.e., 4.4 and 4.1% of the variance for the first and second principal component dimensions, respectively. The MFA analysis of 63 variables identified four components and 16 variables with a correlation coefficient ≥ 0.5 on both first and second dimensions that accounted for 98.7% of the variability in the data (Figure 2). Herd information (ranch and animals sampled) accounted for 27.7% of the total variability in the data, while antimicrobial dosing and record keeping practices (route, dose, date, withdrawal period, and other tracking information) accounted for approximately 25% of the total variability in the data. Nutrition related factors and farm antimicrobial use and disease treatment accounted for 24.2 and 21.6% of the total variability in the data, respectively (Table 5). The sampled animals’ life stage and treatment history as well as antimicrobial resistance data were groups of variables where correlation stayed below the threshold of 0.5.

**Table 5:** Multiple factor analysis (MFA) of 63 data variables collected in a cross-sectional study evaluating antimicrobial resistance status, management factors, and antimicrobial drug use in 18 cow-calf operations in northern California between July 2019 and August 2020. The MFA identified four groups (components) and 16 variables with a correlation coefficient ≥ 0.5 on both first and second dimensions that contribute the most (98.6%) to the variation in the data set.

Identified components	Variation proportion (%)	Component variables	Correlation (*R*^2^)
Herd information	27.7	Sampled ranch	0.99
		Sampled animal	0.99
Nutrition related factors	24.3	Feeding free choice mineral to calves	0.50
		Giving injectable mineral to calves	0.57
		Giving mineral boluses to calves	0.50
		Whether the farm cleans water troughs	0.62
		If they clean water troughs, they use bleach	0.50
Antimicrobial dosing and record keeping practices	25.0	How are antibiotic doses estimated (e.g., estimating weight, standard dose, based on disease, etc.)	0.58
		When an animal is treated, the route is recorded/tracked	0.60
		When an animal is treated, the date is recorded/tracked	0.59
		When an animal is treated, the dose is recorded/tracked	0.57
		When an animal is treated, the withdrawal is recorded/tracked	0.56
		When an animal is treated, other information is recorded/tracked	0.55
		When an animal is treated, nothing is recorded/tracked	0.58
Farm antimicrobial use and disease treatment	21.6	Routine use of antibiotics	0.72
		Use of antibiotics to treat mastitis	0.50

### 3.6. Hierarchical clustering

**Figure 3:**
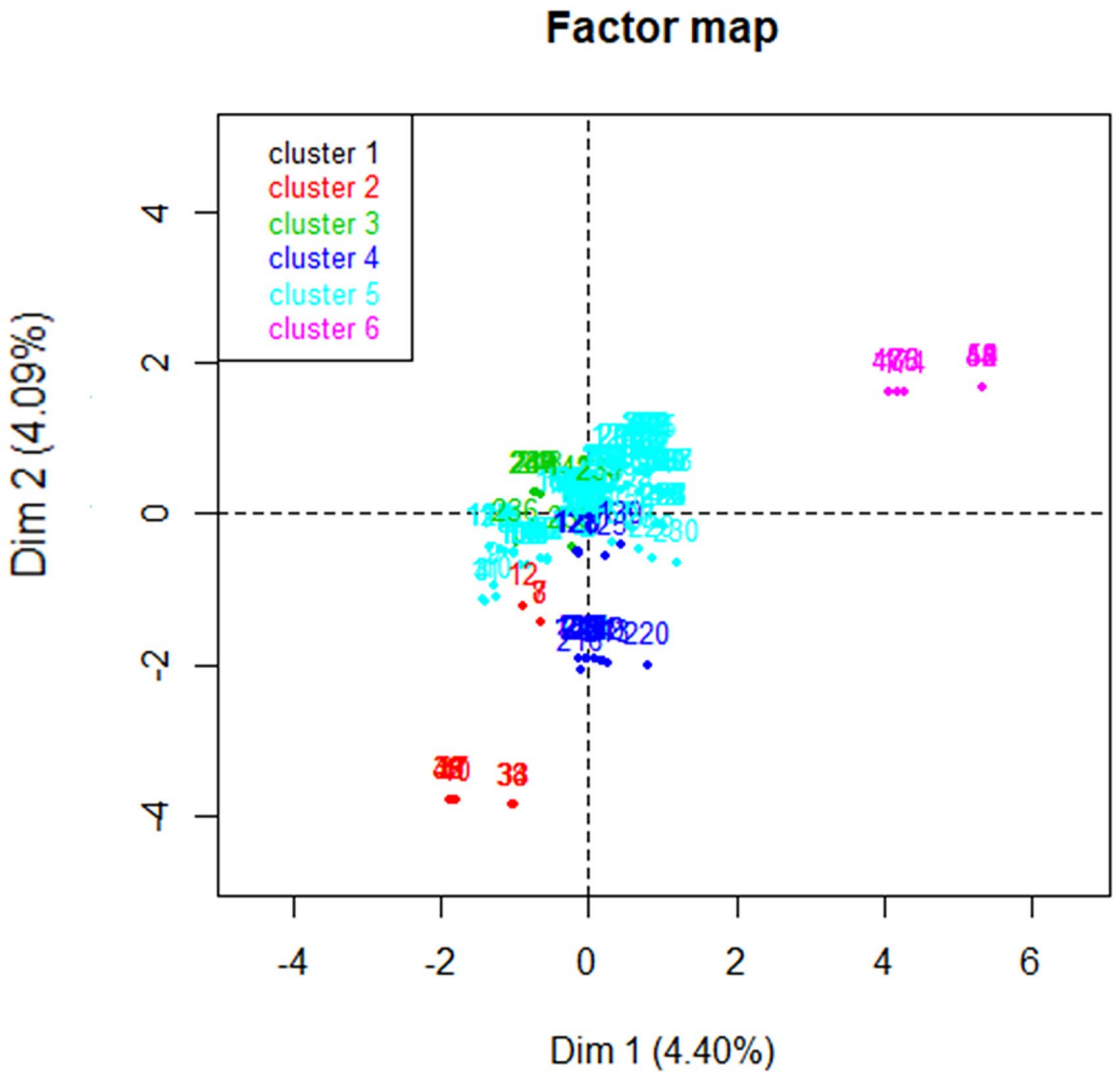
Representation of six clusters identified by hierarchical clustering using the results of multiple factor analysis of data collected from beef ranches in California in a cross-sectional study evaluating antimicrobial resistance and antimicrobial drug use in 18 cow-calf operations in northern California between July 2019 and August 2020.

Hierarchical clustering was performed on the MFA principal coordinates to aggregate homogeneous clusters. The hierarchical tree suggested clustering into six clusters (Figure 3). The identified clusters were described based on the 16 variables that contributed the greatest to the data variability from the MFA analysis (Table 5). Cluster 5 represented the majority (65.2%) of sampled animals and ranches (12/18). Most animals represented in cluster 5 (85.5%) were on ranches that reported estimation of the dose of antimicrobial drugs based on estimated animal weight, reported recording the date of antimicrobial use (91.2%), reported feeding free choice minerals to calves (94.3%), reported cleaning of water troughs (84.9%), and did not use antimicrobials to treat mastitis (100%). However, 91.8% of animals in cluster 5 were on ranches that also reported that withdrawal periods are not recorded when animals are treated with antimicrobials. Cluster 4 represented two ranches in our study. The farms represented in cluster 4 mentioned that they were not recording the date, route, and withdrawal period of antimicrobial use (100%). One farm represented in cluster 2 mentioned routine use of antimicrobials for prevention of disease and use of antimicrobials for treatment of mastitis. Clusters 1, 3, and 6 represented one herd each. Farms represented in clusters 3 and 6 reported that they were not using antimicrobials for treatment of mastitis and the farm in cluster 6 reported dosing antimicrobials according to veterinarian’s orders. The beef operations located in the coastal range were only represented by clusters 5 and 6. The majority of beef ranches in clusters 5 and 6 reported several antimicrobial stewardship or herd health practices including estimation of the dose of antimicrobials based on estimated animal weight, recording of the date of antimicrobial use, feeding free choice mineral to calves, and cleaning of water troughs once a month, and did not use antimicrobials to treat mastitis in comparison to beef ranches included in clusters 1, 2, 3, and 4. A complete description of the six clusters is available in Supplementary Table S1.

## 4. Discussion

Antimicrobial resistance is a global problem (Thanner et al., 2016), and while much of the attentions is focused on human health implications, the effects of AMR on livestock health may be similar, including treatment failures requiring the use of newer and often more expensive antimicrobials (Magnusson et al., 2021).

For our study, the distribution of herd sizes closely represented what has previously been reported for cow-calf operations throughout the state of California (Saitone, 2020). Considering the state’s number of beef cow farms, however, our study included a higher proportion of larger herd sizes for the state, since approximately 77% of beef cow farms in California are reported to have fewer than 100 cows, not including hobby farms with less than 10 cows (United States Department of of Agriculture, 2019). Information about the percentage of different breeds, type of production (organic, natural, No Hormone Treated Certified (NHTC), and/or Age and Source Verified (ASV) versus conventional) for beef cow-calf herds or specific antimicrobial practices have not been previously reported. Due to small numbers in each category, organic, natural, NHTC and/or ASV status were combined to represent how management specific to a target consumer may influence AMR patterns overall. Pasture-based forage is common in California, with livestock grazing being California’s most extensive land use (Saitone, 2020), but details on dryland versus irrigated pasture for beef cow-calf herds have not been reported.

The types of diseases most treated with antimicrobials reported in our survey, namely pinkeye, respiratory disease, footrot and scours, concur with prior data reported in a large survey on antimicrobial use on California cow-calf operations (California Department of Food and Agriculture, 2020). Use of antimicrobials in feed is an uncommon practice in cow-calf herds and mastitis is not nearly as common in beef as in dairy production systems, so it is not surprising that these practices were not common amongst the farms surveyed. In addition, in California, veterinary oversight is required for the purchase and use of all medically important antimicrobials, which may explain the high percentage of farms that reported having a veterinarian-client-patient relationship (17 of 18 farms surveyed).

Of the *E. coli* isolates, approximately 36% were resistant or non-susceptible to at least one antimicrobial, excluding ampicillin, to which all isolates were resistant. AMR of *E. coli* in cattle or ruminants to various antimicrobials has been observed by other authors to varying degrees, but it is not always clear how resistant status is established. For example, one study from Malaysia found 61.9% of *E. coli* isolated from diseased ruminants to be resistant to trimethoprim sulfamethoxazole compared to 4.9% in our study, 69% resistant to tetracycline compared to 13.1% non-susceptible in our study, 54.1% resistant to amoxicillin, compared to 100% resistant to ampicillin in our study (Haulisah et al., 2021). Discrepancies may be due to the choice of breakpoint to establish resistant status, meaning that a breakpoint can be chosen based on the species (human versus veterinary) or it can be chosen based on the most similar bacteria for which there is an established breakpoint, both of which are routine practices and depend on the context of the study. Variations in results can also stem from the fact that diseased animals are more likely to have been treated with antimicrobials before isolation of the pathogen or because antimicrobial drug use patterns may vary between countries or regions.

Ampicillin had the highest proportion of resistant isolates of *E. coli* (100%), which was surprising, especially since none of the participating ranches reported any ampicillin use. In this study, a breakpoint of ≥0.25 μg/mL indicating resistance was chosen based on the veterinary literature for ampicillin resistance for treatment of metritis in cattle due to *E. coli* from VET CLSI [Clinical and Laboratory Standards Institute (CLSI), 2020]. By contrast, the human breakpoint is ≥32 μg/mL indicating resistance. The most common MIC for ampicillin in this study was 4 μg/mL (Table 3). Data from this study indicate that the veterinary breakpoint for ampicillin may need to be reevaluated. The lowest prevalence of resistance or non-susceptibility for *E. coli* of all included antimicrobials was for ceftiofur (0.41%), which was also only used by one of the enrolled farms. Restrictions were placed on extra-label cephalosporin use by the Food and Drug Administration in 2012 which aimed to decrease their use in livestock (FDA Federal Register, 2015).

The ampicillin breakpoint used in this study for *Enterococcus* was ≥16 μg/mL indicating resistance. Although dramatically different from the breakpoint for *E. coli*, this human breakpoint was selected due to available data and differences between antibacterial spectrum and bacterium type. As no veterinary breakpoint is available for this bacterium/antimicrobial combination, the human breakpoint was selected as outlined in the methods, resulting in only 1 resistant *Enterococcus* isolate to ampicillin.

Second to ampicillin, the most common drugs for which *E. coli* was resistant or non-susceptible were sulfadimethoxine and trimethoprim-sulfamethoxazole. A 2016 study of AMR in beef cattle found no associations between the prevalence of resistance of *E. coli* isolates to tetracycline, third generation cephalosporin, or trimethoprim-sulfamethoxazole and history of antimicrobial treatment with either ceftiofur or other antimicrobials (Agga et al., 2016). The authors conclude that mixing of treated and non-treated cattle may mask the effect of treatment or that animal-level effects due to treatment are short-lived. However, both ceftiofur and sulfa treatments were uncommon in our study population so that neither hypothesis would explain the prevalence of AMR to this class of antimicrobial observed.

Florfenicol is a relatively new antimicrobial and limited published, peer-reviewed data exist on resistance profiles. It was first approved for use in cattle in 1998 (Food and Drug Administration Health and Human Services, 1996). In this study, 2 animals had a history of being treated with florfenicol and 9 farms indicated that they use it on farm. For *E. coli*, there were 47 (19.26% of isolate pool) non-susceptible isolates to florfenicol, which was the drug with the third highest AMR prevalence, behind ampicillin and sulfadimethoxine. Reports of increasing AMR to florfenicol in *Enterobacteriaceae* exist in the literature. In addition to antibiotic use, mobile genetic elements and horizontal gene transfer are speculated to play a role in the replication of AMR genes resulting in the observed trend of AMR to florfenicol (Fernández-Alarcón et al., 2011; Li et al., 2020). Future research should also investigate genetic elements linked with phenotypic resistance to AMR to florfenicol in enteric bacteria from cow-calf operations to increase understanding of potential factors resulting in higher prevalence of AMR to this drug.

Historically, bacterial resistance to tetracycline has had a high prevalence (Sato et al., 2004; Gow et al., 2008; Yamamoto et al., 2013). In the present study, *E. coli* and *Enterococcus* isolates had a similar, relatively low proportion of isolates resistant to tetracycline, but *Enterococcus* isolates showed the highest proportion of non-susceptibility to this drug.

No biologically relevant survey variables regarding farm description, animal management, or herd level antimicrobial use were significantly associated with AMR in our models while accounting for correlation between isolates from the same animal. Additionally, controlling for correlation between isolates from the same farm led to unstable models with non-positive G matrices indicating a lack of variation in the additional random effect. However, given the high prevalence of AMR at one of the farms, there may be exposures at farm or animal level associated with AMR that were not captured by this survey.

Calves have been shown to carry more AMR bacteria than cows in previous studies (Berge et al., 2010; Yamamoto et al., 2013; Noyes et al., 2016), however calves in two of those studies were less than 4 weeks old. In contrast, calves in our study were up to one year old, and the bacterial AMR profile in neonates may differ from that of older calves. In dairy calves, antimicrobials may be used more often to treat and prevent disease, but in beef calves, the link is less clear. One hypothesis to explain AMR bacteria shed from calves is that AMR is acquired through other routes, such as genetic linkage (Berge et al., 2010) or direct transfer from cows (Yamamoto et al., 2013) and may not be associated with antimicrobial use on farm.

Another study found the frequency of water trough cleaning and size of operation were significantly associated with AMR prevalence (Fernández-Alarcón et al., 2011). Although water trough cleaning was not significantly associated with AMR in our study, MFA analysis showed both water trough cleaning and whether or not farms used bleach to clean water troughs to be two of the variables contributing most to data variability. Other factors that have been found to be statistically significantly linked to AMR in other studies but were not explored in this study include spring versus fall born calves (Gow et al., 2008) and proximity to dairy farms (Berge et al., 2010). None of the farms in the present study were within one mile of a dairy farm and spring versus fall calves was not examined.

Antimicrobial use on farm has been suggested as a contributing factor for the development of AMR, but several studies have indicated that resistance is multifactorial and develops regardless of exposure or use of particular antimicrobials on farm (Berge et al., 2010; Gaze et al., 2013; Agga et al., 2016), findings that may be substantiated by the results of this study. In addition, there is evidence that some AMR genes may be co-selected or have genetic linkages (Berge et al., 2010; Agga et al., 2016), in which resistance to one antimicrobial is genetically linked to resistance to a different antimicrobial and transferred either vertically or horizontally together (Summers, 2006). Alternatively, antimicrobial use in the cow-calf sector may not exert high enough selective pressures on bacterial populations to drive AMR.

Multiple factor analysis showed that herd information (type of ranch and age of animals) and nutrition related factors (cleaning of water troughs, use of bleach by farmers for water trough cleaning, and provision of mineral supplement to calves) accounted for approximately 52% of the total variance in the data. Several studies have shown herd health in farming systems, herd management, biosecurity, population density, and external pressures to be linked to antimicrobial use (Andrés-Lasheras et al., 2021; Diana et al., 2021). Previous studies reported an association between farm management factors and the prevalence of AMR in *E. coli* isolates (Hille et al., 2017; Markland et al., 2019). Markland et al. (Markland et al., 2019) found that regular cleaning of water troughs and the addition of ionophores to feed were associated with a reduction in prevalence of cefotaxime resistant bacteria in fecal samples of beef cattle on grazing farms in Florida. Beef cattle require several minerals for optimal growth, health, and reproduction. Mineral deficiency may result in anemia, depressed immunity and increased opportunity for bacterial growth and dissemination of resistant bacteria (University of Georgia Extension, 2017). On the other hand, elevated heavy metal supplementation may co-select for antimicrobial resistance of fecal *E. coli* and *Enterococcus* spp. (Jacob et al., 2010). A recent scientific report showed that synthetic smectite clay minerals and Fe-sulfide microspheres have antimicrobial properties and kill antibiotic resistant bacteria including *E. coli* and *Enterococcus* spp. (Morrison et al., 2022) but we did not inquire about the use of these products.

In addition, MFA in this study showed that farm level antimicrobial use, disease treatment, and antimicrobial dosing and record keeping practices accounted for 46% of the total variability in the study data. Similarly, a survey study of antimicrobial use in adult cows on California dairies (Abdelfattah et al., 2019) found that antimicrobial stewardship practices, antimicrobial usage information, and producer perceptions of AMR on dairies accounted for 32.3% of the total variability in the survey data. On the other hand, the sampled animals’ life stage and antimicrobial treatment history and in particular the antimicrobial resistance data contributed to a lesser degree to data variability. Given that AMR seemed less variable than other factors describing the animals and farms in the data set, it is not surprising that statistical models were unable to find associations between AMR and animal or farm related factors. Overall, the MFA analysis identified important differences between herds that can be considered in studies that investigate the risk and the associations between farm practices and AMR of fecal bacteria.

Cluster analysis identified some potential regional differences in management practices and antimicrobial use information among cow-calf operations in northern California since the Coastal Range was only represented by two clusters. The cause of the differences could be due to variable access to information or rancher education or due to the influence of veterinarians in the Coastal Range.

One limitation for this study includes the use of a convenience sample of farms that could have introduced bias because the group of farms that are associated with the University of California teaching hospital or extension agents may have similar management tendencies. They could represent farms that have more progressive management, are more attentive to animal health and/or more willing to treat or may be more likely to adhere to legislation regarding antimicrobial use and antimicrobial stewardship. This is a significant factor to consider and, if true, could have biased the study either toward the null because of less antimicrobial use overall (judicious use) or away from the null because these producers may be more likely to watch carefully, identify, and treat any disease conditions that warrant antimicrobials. In addition to selection of farms, selection of animals for sampling was not random, as sampling is logistically challenging in a cow-calf setting. The animals sampled were either being put through the chute for another reason (processing), were physically closest to the chute, or were the easiest animals to collect for sampling (usually more friendly or animals that are visualized more often in this setting).

Some other challenges associated with sampling in this system include limited animal identification, treatment records, and animal restraint. Many of the animal health records were based on the farmer’s recollection and therefore are subject to recall bias. In this case, those that were identified as treated were very likely actually treated; however, if a treatment was forgotten, that animal did not have any treatment to associate with AMR isolate status. In addition to logistical challenges, none of the farms put antibiotics in the feed which may have biased this study toward the null; however, it should be noted that this practice is not common in cow-calf operations in California. Finally, the use of human breakpoints for the determination of AMR status when no veterinary breakpoints were available is another limitation, underlining the need for further research into AMR in livestock species. A metagenomic analysis of isolates would have provided further information but was not possible at this time due to financial constraints.

## 5. Conclusion

AMR is an evolving, multifactorial topic critical to the health of both animals and humans worldwide. Our study generated novel data for cow-calf AMR, an area with knowledge gaps that limit understanding of factors that could be affecting prevalence of AMR. No associations between specific farm management practices including use of antimicrobials and AMR status of bacterial isolates from the same animals were found and antimicrobial resistance as a variable contributed little to the overall variability in the data; therefore, there are likely other factors that are not well understood and/or not captured in this study that are contributing to development of AMR. In addition, this study presents data to show that antimicrobial use in cow-calf operations in northern California is low, which supports other data in the cow-calf sector. The results of this study serve as a reference for future studies on AMR in the population of cow-calf operations in California and beyond and lead to a better understanding of risk factors for shedding of fecal pathogens carrying ARGs. Continued surveillance will allow more informed decisions and directions for combatting AMR in the future.

## Data availability statement

The raw data supporting the conclusions of this article will be made available by the corresponding author upon request.

## Ethics statement

The animal study was reviewed and approved by UC Davis Institutional Animal Care and Use Committee. Written informed consent for participation was not obtained from the owners because a handout describing the study was provided to owners as well as verbally explained. Interventions on animals were brief and very low risk.

## Author contributions

GM and EO designed the study. DW and GM performed field work and collected data. CM, DW, and EO performed laboratory experiments. CM, RP, EA, and GM analyzed the data. CM and EA prepared the manuscript. EO, EA, RP, and GM revised the manuscript. All authors contributed to the article and approved the submitted version.

## Funding

This research was funded through start-up funds provided through University of California Agriculture and Natural Resources for GM and EO.

## Conflict of interest

The authors declare that the research was conducted in the absence of any commercial or financial relationships that could be construed as a potential conflict of interest.

## Publisher’s note

All claims expressed in this article are solely those of the authors and do not necessarily represent those of their affiliated organizations, or those of the publisher, the editors and the reviewers. Any product that may be evaluated in this article, or claim that may be made by its manufacturer, is not guaranteed or endorsed by the publisher.

## Abbreviations

AMR: Antimicrobial resistance
ARG: Antimicrobial resistance gene
AST: Antimicrobial Susceptibility Test
ASV: Age and source verified
CLSI: Clinical and Laboratory Standards Institute
MDR: Multidrug resistance
MFA: Multiple factor analysis
MIC: Minimum inhibitory concentration
NARMS: National antimicrobial resistance monitoring system
NHTC: Non-hormone treated certified
