# Supplementary material for: Prevalence of antimicrobial resistance in fecal Escherichia coli and Enterococcus spp. isolates from beef cow-calf operations in northern California and associations with farm practices
Source: Front Microbiol. 2023 Feb 23;14:1086203. doi: 10.3389/fmicb.2023.1086203 (PMC9996069; doi:10.3389/fmicb.2023.1086203)
Supplement: Supplementary file 1 [file Table_1.DOCX]

**Supplementary Material**

Table S1: Description of six clusters identified by using multiple factor analysis and hierarchical clustering of a total of 244 *E. coli* isolates obtained from cow and calf fecal samples over 18 cow-calf farms in Northern California collected between July 2019 and August 2020.

|  |  | **Cluster 1** | **Cluster 2** | **Cluster 3** | **Cluster 4** | **Cluster 5** | **Cluster 6** |
| --- | --- | --- | --- | --- | --- | --- | --- |
| **Components** | **Characteristics**  **N (%)** | 16 (6.56%) | 13 (5.33) | 14 (5.74%) | 28 (11.48%) | 159 (65.16%) | 14 (5.74%) |
| **Region** | Coastal Range | 0 | 0 | 0 | 0 | 62 | 14 |
|  | Central Valley | 16 | 10 | 14 | 22 | 62 | 0 |
|  | Northern Central Valley | 0 | 3 | 0 | 6 | 35 | 0 |
| **Herd**  **Information** | **Farm** | | | | | | |
|  | 1 | 0 | 0 | 0 | 0 | 12 | 0 |
|  | 2 | 0 | 0 | 0 | 0 | 18 | 0 |
|  | 3 | 0 | 0 | 0 | 0 | 18 | 0 |
|  | 4 | 0 | 3 | 0 | 0 | 9 | 0 |
|  | 5 | 0 | 10 | 0 | 0 | 0 | 0 |
|  | 6 | 0 | 0 | 0 | 0 | 0 | 14 |
|  | 7 | 0 | 0 | 0 | 0 | 12 | 0 |
|  | 8 | 0 | 0 | 0 | 0 | 26 | 0 |
|  | 9 | 0 | 0 | 0 | 0 | 10 | 0 |
|  | 10 | 0 | 0 | 0 | 0 | 14 | 0 |
|  | 11 | 16 | 0 | 0 | 0 | 0 | 0 |
|  | 12 | 0 | 0 | 0 | 0 | 14 | 0 |
|  | 13 | 0 | 0 | 0 | 0 | 8 | 0 |
|  | 14 | 0 | 0 | 0 | 0 | 6 | 0 |
|  | 15 | 0 | 0 | 0 | 6 | 0 | 0 |
|  | 16 | 0 | 0 | 0 | 22 | 0 | 0 |
|  | 17 | 0 | 0 | 0 | 0 | 12 | 0 |
|  | 18 | 0 | 0 | 14 | 0 | 0 | 0 |
| **Antimicrobial dosing and record keeping practices** | **How are antibiotic doses estimated?** | | | | | | |
|  | Estimate weight | 16 | 10 | 14 | 28 | 136 | 0 |
|  | Weigh animal | 0 | 3 | 0 | 0 | 9 | 0 |
|  | Standard dose by category | 0 | 0 | 0 | 0 | 14 | 0 |
|  | Other | 0 | 0 | 0 | 0 | 0 | 14 |
|  | **When an animal is treated, the route is recorded/tracked** | | | | | | |
|  | No | 0 | 10 | 14 | 28 | 120 | 0 |
|  | Yes | 16 | 3 | 0 | 0 | 39 | 0 |
|  | **When an animal is treated, the date is recorded/tracked** | | | | | | |
|  | No | 0 | 10 | 14 | 0 | 14 | 0 |
|  | Yes | 16 | 3 | 0 | 28 | 145 | 0 |
|  | **When an animal is treated, the dose is recorded/tracked** | | | | | | |
|  | No | 0 | 10 | 14 | 28 | 76 | 0 |
|  | Yes | 16 | 3 | 0 | 0 | 83 | 0 |
|  | **When an animal is treated, the withdrawal is recorded/tracked** | | | | | | |
|  | No | 16 | 13 | 0 | 28 | 145 | 0 |
|  | Yes | 0 | 0 | 14 | 0 | 14 | 0 |
|  | **When an animal is treated, other information is recorded/tracked** | | | | | | |
|  | No | 16 | 13 | 0 | 6 | 145 | 0 |
|  | Yes | 0 | 0 | 14 | 22 | 14 | 0 |
| **Nutrition Related**  **Factors** | **Does the farm feed free choice mineral to calves?** | | | | | | |
|  | No | 16 | 0 | 0 | 0 | 0 | 0 |
|  | Yes | 0 | 10 | 0 | 22 | 150 | 14 |
|  | **Does the farm give injectable mineral to calves?** | | | | | | |
|  | No | 0 | 13 | 0 | 22 | 91 | 0 |
|  | Yes | 0 | 0 | 14 | 6 | 68 | 14 |
|  | **Does the farm give mineral boluses to calves?** | | | | | | |
|  | No | 0 | 13 | 0 | 28 | 125 | 14 |
|  | Yes | 0 | 0 | 14 | 0 | 34 | 0 |
|  | **How often does the farm clean water troughs?** | | | | | | |
|  | Never | 0 | 0 | 0 | 6 | 24 | 0 |
|  | < once a month | 0 | 13 | 14 | 0 | 135 | 14 |
|  | > once a month | 0 | 0 | 0 | 22 | 0 | 0 |
|  | **If they clean water troughs, do they use bleach?** | | | | | | |
|  | No | 0 | 3 | 14 | 22 | 135 | 14 |
|  | Yes | 0 | 10 | 0 | 0 | 0 | 0 |
| **Farm level antimicrobial use and disease treatment** | **Does the farm have routine use of antibiotics?** | | | | | | |
|  | No | 0 | 3 | 14 | 28 | 159 | 14 |
|  | Yes | 0 | 10 | 0 | 0 | 0 | 0 |
|  | **Farm uses antibiotics to treat mastitis** | | | | | | |
|  | No | 16 | 3 | 14 | 6 | 159 | 14 |
|  | Yes | 0 | 10 | 0 | 22 | 0 | 0 |
| **E. coli AMR** | **Ceftiofur resistance** | | | | | | |
|  | No | 16 | 13 | 14 | 27 | 157 | 14 |
|  | Yes | 0 | 0 | 0 | 0 | 1 | 0 |
|  | **Florfenicol non-susceptibility** | | | | | | |
|  | No | 15 | 8 | 11 | 27 | 157 | 6 |
|  | Yes | 1 | 5 | 3 | 1 | 2 | 8 |
|  | **Sulfonamide resistance** | | | | | | |
|  | No | 15 | 6 | 9 | 23 | 124 | 5 |
|  | Yes | 1 | 7 | 5 | 5 | 35 | 9 |
|  | **Tetracycline non-susceptibility** | | | | | | |
|  | No | 16 | 7 | 9 | 25 | 154 | 6 |
|  | Yes | 0 | 6 | 5 | 3 | 5 | 8 |
|  | **Sulfadimethoxazole resistance** | | | | | | |
|  | No | 16 | 13 | 14 | 27 | 156 | 6 |
|  | Yes | 0 | 0 | 0 | 1 | 3 | 8 |
